# A comparative study of frequency effect on acquisition of grammar and meaning of words between Chinese and foreign learners of English language

**DOI:** 10.3389/fpsyg.2023.1125483

**Published:** 2023-07-25

**Authors:** Jizheng Zhao, Jing Huang

**Affiliations:** ^1^School of Foreign Languages and Business, Shenzhen Polytechnic University, Shenzhen, Guangdong, China; ^2^Department of English Language Education, Education University of Hong Kong, Hong Kong, Hong Kong SAR, China

**Keywords:** frequency effect, grammar, meaning, alphabetic, hieroglyphic, second language acquisition (SLA)

## Abstract

Frequency effect on vocabulary acquisition has been widely investigated in second language acquisition (SLA) research, whereas comparative studies of vocabulary acquisition of learners from different language types, such as hieroglyphic writing and alphabetic writing, are still rarely found. This type of studies could be of great significance in exploring some unique characteristics of how second language learners of native languages of different writing perceive and acquire second language. Using artificial words of alphabetic writing and low-frequency English words as experimental materials, this study aims to compare the effect of frequency on the acquisition of grammar and meaning of alphabetic words between Chinese learners of the hieroglyphic native language and foreign learners of alphabetic native languages. Specifically, the study intends to find out whether frequency effect plays the key role in language acquisition; to what extent frequency effect affects language acquisition; and whether there are any differences between learners of different language types for vocabulary acquisition in terms of frequency effect. The results show that Chinese and foreign learners of English language have no significant differences as a whole in terms of type of languages affecting the acquisition of grammar and meaning of artificial words and English words, indicating the difference in the type of mother tongue might not be the factor causing differences on grammar and meaning acquisition of vocabulary. Learner types, language types, frequency and part of speech of a word have interaction effect toward the acquisition of grammar and meaning of a word. However, exposure frequency of vocabulary plays the determining role in the acquisition of grammar and meaning of words.

## Introduction

Many researchers (Larsen-Freeman, 1997; MacWhinney, 1999; Ellis, 2002a,b, 2008; Schwartz and Causarano, 2007) claimed that frequency has a major role in second language acquisition. Ambridge et al. (2015) indicated that frequency effects were ubiquitous in virtually every domain of human cognition and behavior, from the perception of facial attractiveness (Grammer and Randy, 1994) and the processing of musical structure (Temperley, 2007) to language change (Bybee, 2010) and adult sentence processing (Ellis, 2002a). Ambridge et al. (2015) claimed that frequency effects are pervasive in children’s first language acquisition and argued therefore that any successful account of language acquisition, from whatever theoretical standpoint, must be frequency sensitive to the extent that it can explain these effects. Ellis and Ogden (2015) further emphasized that the same conclusion follows from 60 years of psycholinguistic research into the fluent language processing that culminates from acquisition: Language processing is exquisitely sensitive to usage frequency at all levels of representation, e.g., phonology and phonotactics, reading, spelling, lexis, morph syntax, formulaic language, language comprehension, grammaticality, sentence production, and syntax.

Ellis (2002a) pointed out that the frequency effect, after 40 years of exile, returned to researchers’ focus again. According to Leech (2011), three theoretical positions that have been gaining momentum since the 1990s, all implicitly or explicitly give frequency a role in the workings of language: usage-based linguistics, cognitive linguistics, and construction grammar. Ellis (2002a) believed that language acquisition is cumulative example-based learning of thousands of constructions, as well as a frequency-based process of abstracting internal rules. Regularities of language emerge when learners are exposed to categories and prototypes. The frequency effect plays an important role in explaining sociolinguistic variants and language changes.

Andringa and Rebuschat (2015) held similar ideas for the role of frequency in language acquisition, indicating that statistical learning is an incremental accumulation of language knowledge on the basis of input distribution characteristics, with learners being very sensitive to the input distribution characteristics. A significant characteristic of statistical learning is that it will emerge automatically and unconsciously when people are exposed to language input.

In fact, since the late 20th century, many theories in the linguistics sphere, including implicit learning theory (Ellis, 1994, 2002a), dynamic system theory (DST) (Larsen-Freeman, 1997, 2007; Verspoor et al., 2008), construction grammar theory (Langacker, 1987), connectionism (Rumelhart and Melland, 1986, 1987), emergentism (Elman et al., 1996; Ellis and Schmidt, 1998; Ellis and Larsen-Freeman, 2006), and usage-based language theory (Bybee, 2006; Zhao, 2017), which are from the same theoretical paradigm and inextricably related, tend to support the view that language learning mechanism is not different from the other cognitive mechanism that language is acquired by using.

Besides, embodied philosophy represented by Lakoff and Johnson (1999) also emphasized that human language is derived from language use and formed through the interaction of brain, body and the environment. It proposed a theory of body-mind unity and internal-external unity (unity of man, nature and society) which provides a good foundation for taking some other factors into consideration.

Despite the fact that research on frequency effect on SLA has been extensively carried out over decades, comparative studies between EFL learners of different language types remain scant. What role does exposure frequency play in target vocabulary acquisition? Could it be possible that the difference of writing forms of native languages has different frequency effect on vocabulary acquisition of the target language? Does exposure frequency interact with the other factors, such as learner type, language type and the grammar of a word, in affecting target vocabulary acquisition? Adopting an experimental design (Creswell, 2012; Creswell and Guetterman, 2019), the present study set out to explore all these questions.

## Literature review

Harrington and Denis (2002) classify the frequency effect as “an attribute of individual experience” and “an attribute of the linguistic environment.” The former is called “task frequency,” and the latter “distribution frequency.” This study mainly focuses on task frequency, at which a learner is exposed to a linguistic item.

### Frequency rates and vocabulary acquisition

For the purpose of determining what might be the “most” appropriate exposure frequencies for vocabulary acquisition, researchers have carried out extensive studies on the frequency effect on this from different perspectives. Studies on the correlation between exposure frequency and the acquisition and retention of vocabulary are one of the perspectives that some researchers focus on. Saragi et al. (1978) showed that the correlation between vocabulary acquisition and vocabulary frequency was 0.34, which presents the frequency effect on learning but indicates that learners need to be exposed to a word 10 or more times (a common number for mastering vocabulary knowledge) before achieving a significant effect on vocabulary knowledge acquisition. Horst et al. (1998, p. 215) showed that the correlation coefficient between exposure frequency and acquisition is 0.49, indicating at least 8 or more times of exposure required for vocabulary acquisition; that is, with fewer than 8 times of exposure, the acquisition effect would be difficult to predict. The study found that notional word acquisition had a higher acquisition score, and the images had a significant effect on the acquisition. The study by Waring and Takaki (2003) found that learners could have a 50% probability of recall and comprehension of a word 3 months later if they were exposed to a word in the target language at least 8 times. If the learners were exposed to the word 18 times, it was likely for them to remember the meaning of the word after 3 months. Therefore, they recommended that learners be exposed to a new word more than 20 times for acquisition. Rott (1999) studied the effects of vocabulary exposure frequency on vocabulary acquisition and retention in the mid-level language learners’ reading process. The results showed that the students exposed to the words (2, 4, or 6 times) were significantly better at mastering vocabulary knowledge than the students who were not exposed to the words; both 4 and 2 exposures resulted in no significant changes in the acquisition of vocabulary input and output knowledge, while 6 exposures had a significant effect on the acquisition of the two kinds of knowledge compared with 4 exposures.

Studies on exposure frequency and acquisition of various knowledge of vocabulary are another perspective that researchers focus on. Webb’s (2007) study focused on the frequency effect and seven aspects of vocabulary knowledge. Learners are exposed to the words 1, 3, 7, or 10 times. The results showed that each exposure to a word increased knowledge in at least one variable. If learners are exposed to the word 10 times, they acquire objective word knowledge. The mastery of word knowledge, however, may require more than 10 exposures. Chen and Truscott (2010) studied whether L1 lexicalization affects vocabulary acquisition. The lexicalized words referred to words having equivalent translations and fixed linguistic items in Chinese, while the non-lexicalized words had no equivalent translations in Chinese. The results showed that the frequency of exposure to lexicalized words had a greater effect than non-lexicalized words in acquisition, demonstrating that the number of exposures had a positive and significant impact on learning. However, for the acquisition of non-lexicalized words, both the immediate post-test and the delayed post-test showed reduced acquisition. Even after 7 exposures, it is still impossible to acquire non-lexicalized words.

In addition, Sun (2014) examined the effect of contextual richness on vocabulary acquisition and studied the relationship between frequency and vocabulary knowledge acquisition (see also Webb, 2007). Zhang and Qi (2009), using natural authentic reading materials, studied incidental vocabulary acquisition. Song and Sardegna (2014), focusing on the grammatical perspective, studied the frequency effect on the acquisition of English prepositions and showed that frequency exposure to propositions of various contexts and learners’ participation in output activities could help to acquire the target features. Aka (2019) investigated the frequency effect on the acquisition of the grammar structure of to-infinitive used as noun, indicating frequent exposure to target grammar items repeatedly helps learners notice a grammatical rule and will contribute positively to grammar acquisition. Zhang (2020) finds that the frequency effect on the processing of formulaic sequences by Chinese native speakers is significant. Zhang and Zhang (2022) studied the developmental features of the receptive-productive continuum of L2 academic vocabulary, and the results showed that there was a significant positive correlation between subjects’ overall proficiency of academic vocabulary and the frequency level of the corresponding vocabulary. Although the character of frequency in this study is distributive, the researchers emphasized that a higher distributive frequency vocabulary meant more probabilistic and contacting opportunities in input and that the vocabulary had more chances to be activated.

### Embodied cognition and SLA

To make the present study more justified, this research has also taken into consideration the interplay of various internal and external factors. Atkinson (2010, p. 599) stated:

*…conceptions of cognition have changed radically over the past century…….toward extended and embodied views of cognition. Extended cognition conceptualizes mind/brain as inextricably tied to the external environment, while embodied cognition views cognitive activity as grounded in bodily states and action. These two approaches are related because bodies link minds to the world–we experience, understand, and act on the world through our bodies.*

Boden (2006) indicated that instead of being the self-contained logical system posited by cognitivism, cognition depends heavily on the external environment. Atkinson (2002) developed the notion of sociocognitive perspective on SLA and advocated that language and language acquisition as simultaneously occurring and interactively constructed both “in the head” and “in the world” (p. 525).

According to embodied psychology and language cognition (Wang, 2012), language acquisition should focus on the integration of language cognition and the physical and external environment, as well as the role that the body and the environment play in the cognition process. The theory pays more attention to the physical body, the local environment (situation) and the interaction of the nerve system with the corresponding external environment. Human brain, body and environment are constantly changing and interacting. The true cognitive system is a unified system consisting of all three. Therefore, a common point of embodied philosophy and cognitive linguistics is that categories, concepts, reasoning, and thought of human beings are formed through people’s physical experience, and language is gradually formed through people’s cognitive processing relying on the interaction of the sensory organs with the real world. Language acquisition is the result of multiple interactions between subject (human being) and object (environment). These ideas are in line with Atkinson’s (2010, p. 612) sociocognitive view of SLA:

*…sociocognitive approaches to SLA are based on this tripartite premise: (i) Mind, body, and world are in continuous processes of interaction alignment; (ii) These processes are partly public; and (iii) In being public, they are learnable. Thus, if cognition is the site of learning, it is extended, embodied cognition that makes learning possible, at least in part.*

Besides, embodiment theories are regarded as being capable of complementing usage-based approaches and should be incorporated into existing L2 theories (Patterson, 2021). Using usage-based and embodiment approaches, Patterson (2021) investigated second language listening functor (function words) comprehension probability. Transcription of functors were used as the dependent variable and frequency, word length, and Minkowski3 sensorimotor ratings as independent variables. The results showed that greater frequency, longer word length, and higher Minkowski3 ratings were found to facilitate comprehension and significantly increase the probability that a functor was transcribed. Frequency rates derived from spontaneous L1 oration and conversations were found to be significant.

### Usage-based approach and frequency effect

In fact, usage-based approach is widely used in frequency effect studies of SLA (Bybee, 2006; Ellis et al., 2008; Ellis and Larsen-Freeman, 2009). Usage-based linguistics argues that language acquisition takes place through implicit learning (using cognitively generic learning strategies) of patterns of form and meaning encountered in language input. Ellis and Ogden (2015, p. 283) held the view:

*Learning, memory, and perception are all affected by frequency of usage: the more times we experience something, the stronger our memory for it, and the more fluently it is accessed. The more recently we have experienced something, the stronger our memory for it. The more times we experience conjunctions of features, the more they become associated in our minds and the more these subsequently affect perception and categorization; so a stimulus becomes associated to a context and we become more likely to perceive it in that context.*

Usage-based linguistics thus recognizes the impact of language usage on language cognition representation. It emphasizes that as users are exposed to language tokens, they classify their forms in different abstract forms. This classification process forms a network that includes speech, semantics, and pragmatics. This type of network is subject to language frequency. The usage-based language theory actually regards language knowledge as a set of automatic, generalized sentence patterns.

In terms of the influence of frequency on SLA, the usage-based theory holds that: (1) L2 language learners find it difficult to learn language because of a lack of a mother tongue acquisition environment; (2) L2 learners have the comparison mechanism for language decoding and output as well as the mechanism for linguistic and non-linguistic classification. These mechanisms can be used for acquiring new languages. The only requirement for L2 learners is to have sufficient exposure to second language (L2). At the same time, chunking and automaticity processes require a wealth of links between language and non-language to reach fluency.

### Studies using samples of different language types

Studies of this type could be of great importance for exploring unique characteristics such as how second language learners of different native languages perceive and acquire second language; how different related factors affect SLA; what role exposure frequency plays in the acquisition process; and whether frequency may have any universal effect on SLA for learners of different native languages. Therefore, research in this line, may involve learners of different native languages, learners’ native cultures and life environments, their perception of different languages, the interaction of linguistic and non-linguistic factors while learners are learning a new language.

The study by Chen et al. (2020) might be one of the few comparative studies on the frequency effect on SLA of learners of different native language types. Using artificial words of alphabetic writing as experimental materials, they investigated the acquisition of alphabetic word forms between Chinese learners of the hieroglyphic native language and foreign learners of the alphabetic native language. The results showed that the difference in the character pattern of the mother tongue could result in disparity of the acquisition rate of the character pattern, and the word acquisition rate of the same character patterns was higher. The results also showed that input frequency could go beyond the difference of mother tongue and shared some common features, indicating that the frequency of being exposed to the language can overcome and transcend the barriers of language differences during language acquisition.

Perez-Paredes and Bueno-Alastuey’s (2019) study consisted of subjects of native speakers (NSs) and Non-native speakers (NNSs) of Chinese, German and Spanish. The research explored the most frequent certainty adverbs in the extended LOCNEC and their frequency and use in three datasets of the LINDSEI (Chinese, German, and Spanish LINDSEI components). The study yielded a complex picture and no simple rule could be drawn from the data on the use of stance adverbs by learners of different native languages. An important finding relevant to the present study is that NSs and Chinese frequencies of use for adverbs were not significantly different. The researchers believed that this might be attributed to that the two groups approached the task in ways different from the German and Spanish speakers. In this study, an examination of the pragmatic contexts of using the certainty adverbs revealed that both NSs and NNSs restricted their semantic choice to classic epistemic meanings with few instances of more complex pragmatic meanings. Complex might be the results of this study, we can still find that the learners, in spite of the differences of their native languages, share more similarities than differences in using the target language.

Ament et al. (2020) explored the distribution of pragmatic marker (PM) use by English as a Foreign Language (EFL) speakers and English native speakers (NSs). Participants were second-year (N1/423), and third-year (N1/418) business undergraduates, and a NS control group (N1/410). Via English-medium instruction (EMI), the researchers increased learners’ contact with English to explore the use of textual PMs in their oral communication. The results indicated that the EMI groups used PMs for causal, contrast and sequential functions at similar frequencies as NSs, and that the NSs used PMs significantly more often for continuation and elaboration functions and significantly less opening and closing functions compared to the EMI groups. The study suggested that EMI may play an important role in facilitating the acquisition of some functions of PMs, whereas other PMs, such as elaboration markers, may take longer to acquire.

Zhang and Fang (2020) investigated frequency effect on collocation processing of native speakers of English (NSs) and Chinese EFL learners (NNSs). Same-translated collocations and different-translated collocations were chosen as the experimental materials. Online acceptability judgment task of English collocations was used to measure subjects’ performance. The study showed that both NSs and NNSs processed more accurately same-translated collocations but not faster than judging different-translated collocations; NNSs’ language proficiency modulated the effects of constituent word frequency and collocational frequency on the processing output; and lexical frequency played a modulating role in the processing of all types of collocations for both NSs and NNSs. The results indicated that the ultimate goal of second language learners was to infinitely approach the overall processing of collocations in the native language. Zhang and Fang (2020) assumed that frequency is the determining factor for collocation acquisition, and frequency of exposure is ultimately experience, an example, and the use of language. In terms of frequency effects, there is no bipolar debate between NSs and NNSs; it is a gradual transition and evolution from dependence on rules to overall synthesis as the frequency of contact increases and language proficiency grows.

Based on the thorough review of related literature in this section, we may draw the following conclusions: (1) Exposure frequency plays an important role in language acquisition. (2) The human being’s body and brain may interact with the environment and unify to affect language acquisition. (3) Learners have language processing mechanism which can equip them with the ability to distinguish linguistic and non-linguistic factors. While learners expose themselves to the input-rich environment in which linguistic factors and non-linguistic factors interact constantly, they are supposed to be able to achieve language fluency. Therefore, (4) learners’ native languages and cultures, learners’ mental and physical factors, learning contexts and environments, the interaction between learner physical condition and environment, the interaction of linguistic and non-linguistic factors, are all possible factors affecting SLA. (5) Comparative studies on frequency effect of different language types toward second language vocabulary acquisition are still at its initial stage, and therefore, more studies in this line are needed.

## Research questions

This study wants to continue with the line of comparative research on vocabulary acquisition and concentrates on the acquisition of grammar and meaning of words between learners of different language types. The purpose of this study is to find out whether frequency effect, among all the factors relevant to SLA, plays the key role in language acquisition and to what extent it affects language acquisition; and whether frequency exposure of language might have some universal effect on SLA across learners of different cultures.

This study assumes that language could be a symbolic icon representing culture. People’s value and perception of the world could be embedded in languages and they may affect the process of SLA implicitly. Therefore, in this experiment we choose two types of learners of English whose native languages are very different in forms: Chinese learners of hieroglyphic writing and non-native speakers of alphabetic writing. To avoid culture bias for Chinese learners of hieroglyphic writing, and foreign learners of alphabetic, a language which could both symbolize alphabetic writing and without much cultural embodiment might be an appropriate choice as one of the target languages, for which an artificial Keki language (McCandliss et al., 1997) is chosen. Besides, the study also aims to investigate what the situation might be while learners acquire a real language. Hence, the study uses the low-frequency words in high-frequency category of Corpus of Contemporary American English (COCA), for the purpose of both symbolizing the form of real alphabetic language to the greatest extent, and with the least possibility of representing its meaning.

The study addresses the following research questions (RQs):

*RQ1. Do Chinese and foreign learners of English differ in the frequency effect on the acquisition of grammar and meaning of artificial words and English words?*

*RQ2. What is the general role of frequency in Chinese and foreign learners’ acquisition of grammar and meaning of artificial and English words?*

*RQ3. Does the interaction of factors such as learner type, language type, frequency and part of speech influence lexical acquisition?*

*RQ4. Does the acquisition of grammar and meaning of words vary in accordance with the difference in language type?*

## Materials and methods

### Subjects sampled for the experiment

To maintain the validity of the experimental data, we chose 30 subjects for both Chinese group and foreign group. And 30 subjects are regarded sufficient for a psychological experiment (Chen, 2005, p. 43). The following criteria were established to keep the homogeneity of the subjects, including (1) all subjects had not previously lived or studied in countries of native English language; (2) they were all tertiary level students, and their English proficiency should be at the same level; (3) they all had normal visual acuity (corrected or uncorrected). The second-year Chinese students of English major whose native tongue is hieroglyph and foreign students whose native language is alphabetic writing were the two target subject groups. Both the Chinese learners and foreign learners were in a same university in southern China.

To keep consistent the proficiency level of the participants, we invited the English teachers of these two target groups to evaluate these students’ English level, based on these students’ formative achievements (e.g., quiz scores) and the results of semester final examination to eventually choose 60 Chinese and 60 foreign volunteer students of similar English proficiency level as the candidate participants. Further, we adopted College English Test Band 6 (CET6) July 2020 as the tool to test the subjects’ English proficiency. Given that some subjects are foreign students, the sections of Writing and Translation were eliminated and only sections of Listening (total score = 248.5) and Reading (total score = 248.5) were kept. The testing procedures of these two sections were strictly implemented as required in the real test. The results showed that there was no significant difference in terms of English proficiency between the two groups (*T* = 0.075, *P* = 0.092). We sampled 30 subjects from each group whose test results were among the middle range (*M* = 388 for the Chinese group; *M* = 392 for the foreign group) of the 60 candidate participants in each group. The foreign group consisted of students from Russia, Republic of Korea, Kazakhstan, Slovakia, and Italy.

### Ethical considerations

The experiment was conducted with the participants’ informed consent. Before the experiment, we explained the purpose and content of the experiment to the participants. We emphasized that different national and ethnic cultures and different writing forms of their native languages would be used for academic purpose only, without prejudice against any specific cultures and writing forms. We respected the freedom of participants and allowed them to withdraw from the experiment as they wish. All possible measures were taken in the experiment to ensure that participants did not experience any adverse reactions due to their participation in the experiment. The participants were informed that their performance during the experiment and the results of the experiment would be kept strictly confidential. They would be awarded Y– 40 each for participation.

### Experimental materials

#### Artificial words and low frequency English words

Two language types are chosen for the experiment: artificial Keki language and English language. The artificial Keki language includes 68 artificial words (see Supplementary Appendix 1). The composition of the words follows the rules of C(C)VC(C)V (C stands for consonant, V vowel). There are many similarities in composition between Keki and English words; the only difference is that the words in Keki end in vowels. The uniqueness of Keki words means that they have some features of English spelling but are different from English. These features can properly reflect the characteristics of alphabetic writing with no similarities to hieroglyphics and therefore have favorable representation and test validity for measuring differences in words between alphabetic writing and hieroglyphics. The materials consist of two parts, one of which is used in the learning phase and the other in the test phase. Part 1 materials in the learning phase include artificial words (see Supplementary Appendix 2) and low-frequency English words (see Supplementary Appendix 3). Artificial words are from the artificial Keki language. We chose the target artificial words according to the parts of speech of target words needed for the experiment. Therefore, some more artificial words were created based on the word formation rules of the Keki language to satisfy the requirement of experiment. The experimental materials include 4 groups of notional words; each has 6 words, including 2 nouns, 2 verbs, and 2 adjectives, for a total of 24 words. The exposure frequencies of the four groups of words were 1 time, 3 times, 7 times, and 10 times. The corresponding learning materials are low-frequency English words. The grouping form, part of speech in the groups, and frequency of words presented are identical to the artificial words.

The 24 English words in the four groups of the experiment are low-frequency words selected from the sampling list of COCA, extracted from every 7 words in the list of the top 60,000 high-frequency words in the COCA corpus, and including a total of 8,574 words. On this basis, we select 24 notional words from back to front in the list. Therefore, the 24 English words used in the experiment are low-frequency words in the 60,000 high-frequency words in the COCA corpus. Such selection can both satisfy learners’ perception of high-frequency words and ensure that students probably have not been exposed to such words to the greatest extent possible.

The reason we use artificial words and English vocabulary at the same time in the experiment for meaning and grammar lies in the fact that the artificial Keki language has the characteristic of alphabetic writing, but it is different from English, which has a certain neutrality. For Chinese learners of hieroglyphs, Keki language has similarities with English language in its form. For foreign students of alphabetic writing, it is similar to their native language, as well as English. Furthermore, the critical difference between the artificial words and real words lies in that the former is assumed not containing cultural information, while the latter contains cultural information. The use of such artificial words can commendably test the real condition of learners of English from hieroglyph and alphabetic backgrounds on the grammar and meaning of alphabetic writing. For the adoption of English to experiment on Chinese and foreign learners, its purpose is to test, in real language, whether there is a difference between them in the acquisition of grammar and the meaning of vocabulary and whether it differs from the grammar and meaning of vocabulary of the artificial language. If differences exist and are distinct, it is suggested that the word form difference of characters might also be an important reason for the acquisition difference of grammar and meaning of vocabulary of Chinese and foreign learners with different word forms in their mother tongues, and the language environment and cultural differences behind vocabulary acquisition are worthy of study.

Part 2 materials of the experiment are testing materials, including test questions of artificial words (see Supplementary Appendix 4) and English low-frequency words (see Supplementary Appendix 5). Sentence completion with multiple choices is adopted. There is one space in each sentence, which requires the subjects to choose one right answer from the four options and fill in the blank. The four options include distractors developed based upon misconceptions of word meaning and language type and with special attention given to grammar. For example, test on the artificial word *gonta*:

##### gonta

*She seems very gonta with the result, for that is all what she can do.*

*A. sad B. happy C. greatly D. gone.*

Among the four options, two adjectives, including “sad” and “happy,” one adverb, “greatly,” and one past participle “gone,” are included. When presenting the target words in E-Prime, apart from the meaning of the target words, the grammatical element–part of speech of the word–is also presented, such as “gonta, adj. happy.” Coupled with the connection of the target word with the word before and after it in the sentence, learners make a judgment: an adjective should be chosen for this space, and its meaning should be happy. Therefore, the choice of right answer not only requires the subjects to infer the meaning but also the part of speech of the target word.

#### Measurement of grammar and meaning of vocabulary

The grammar of vocabulary involved in this experiment is presented through the part of speech of words. Considering the distribution of part of speech of words in language and in avoidance of the impact of close-class words such as articles and prepositions on the test results, three types of open-class words–noun, verb and adjective–are selected for this experiment. The accuracy of words with different parts of speech selected by the subjects in the experiment can be regarded as the acquisition rate of the grammar knowledge of words by them. The accuracy of acquisition of the meanings of words is determined by the correctness of the meanings of words selected by the subjects. In other words, if learners select the correct answer during the test period in the experiment, it is supposed that they have mastered the grammar and meaning of such words. Therefore, in this experiment, the accuracy of acquisition of grammar and meaning of different language types is determined by selection of the right answers from multiple choices.

### Experimental design

Factorial design (Creswell, 2012, p. 311) was adopted and a multi-factor mixed design was created. Four factors used as independent variables are learner type, language type, part of speech of the target word and exposure frequency of target words. Factor as dependent variable is subjects’ achievements in the test task. The purpose of this design is to test the main effect of frequency and acquisition and at the same time the interaction effect of all the factors for acquisition. Specifically multi-factor 2 × 2 × 3 × 4 mixed design is used in the experiment. Independent variable 1 (variables between subjects) is learner type (two groups: foreign students from non-native English-speaking countries and Chinese learners of English); independent variable 2 (within subjects) is language type (artificial language and English language); independent variable 3 (within subjects) is the part of speech of the target word (adjective, noun and verb); and independent variable 4 is the exposure frequency of target items, including four frequencies in total (1 time, 3 times, 7 times, and 10 times). In the experimental design, repetition within and between the frequencies of words was avoided. In addition, words of the four frequencies were presented randomly, which aimed to prevent students from feeling tired or guessing the answers during the learning and testing period. The dependent variable is tested by using multiple choice questions. According to the number of target words, we designed six sentences in which the target words are underlined and four options are provided for each question. Other than the test on understanding of meanings, distractors are also designed to test the subject’s grammar knowledge. A score of 5 points is assigned for each question, 5 points for a right choice and zero for a wrong choice. Therefore, the full score for the target word of each part of speech is 10 points for each exposure frequency. The full score of the four frequencies is 120 points. The score is counted through a computer.

### Experimental procedure

Learning-recognition paradigm is widely used in psychological experiments (e.g., Liao and Zhang, 2012; Zhang and Xing, 2012). The procedure is divided into two steps: “learning” and “recognition”; “recognition” is immediately implemented after the subjects complete the task at the “learning” stage.

The learning and recognition steps are computer-based and programmed and recorded by E-prime. In the experiment, after one level of exposure frequency (1, 3, 7, or 10) of words, the recognition test is carried out immediately. During the learning period, the instruction is first presented to the two subject groups of Chinese and foreign learners, which is shown in both Chinese and English.

When presenting the target word, three types of information are shown in each page on the screen–target word, part of speech and meaning of the target word. Before the presentation of each artificial word, a string of “*” is shown on the screen, lasting for 500 ms, priming the subjects’ attention, followed by artificial words presented, lasting for 8 s for each word without an interval. The subjects rest for 10 s after learning a sequence.

After the break, the subjects come to the recognition test phase for the experiment. The presentation time for each word is 15 s without an interval. The recognition test is conducted immediately after learning.

## Results

According to the questions under study, we sorted the experimental data and performed statistical analysis with SPSS17.0. Four copies of invalid test materials in each group in the experiment were rejected; 26 copies of valid test materials in each group were used for analysis. To learn the overall situation of the acquisition of target words in the sentence context of Chinese and foreign learners, we performed descriptive statistics. The acquisition of artificial words and English words is shown in Tables 1, 2, respectively.

**TABLE 1:** Acquisition mean of artificial words with different parts of speech at different frequencies of Chinese and foreign learners.

	Learner type	Case number	Mean	Std. deviation	Mean of std. error
A1adj	Foreign learner	26	8.08	3.187	0.625
	Chinese learner	26	6.92	3.486	0.684
A1n	Foreign learner	26	7.50	3.240	0.635
	Chinese learner	26	6.54	3.679	0.722
A1v	Foreign learner	26	7.50	3.240	0.635
	Chinese learner	26	6.54	4.188	0.821
A3adj	Foreign learner	26	8.27	3.144	0.617
	Chinese learner	26	7.12	3.788	0.743
A3n	Foreign learner	26	9.23	1.840	0.361
	Chinese learner	26	7.31	3.803	0.746
A3v	Foreign learner	26	6.92	4.019	0.788
	Chinese learner	26	6.92	4.019	0.788
A7adj	Foreign learner	26	9.62	1.961	0.385
	Chinese learner	26	8.46	3.397	0.666
A7n	Foreign learner	26	9.42	1.629	0.319
	Chinese learner	26	9.04	2.010	0.394
A7v	Foreign learner	26	8.65	3.019	0.592
	Chinese learner	26	8.46	3.397	0.666
A10adj	Foreign learner	26	8.46	2.746	0.538
	Chinese learner	26	8.65	3.019	0.592
A10n	Foreign learner	26	9.04	2.835	0.556
	Chinese learner	26	9.04	2.835	0.556
A10v	Foreign learner	26	9.04	2.457	0.482
	Chinese learner	26	8.27	2.426	0.476

Number (1,3,7,10) = frequency (1,3,7,10); A, artificial; adj., adjective; n, noun; v, verb.

**TABLE 2:** Descriptive statistics of Chinese and foreign learners’ acquisition of English words of different parts of speech under different frequencies.

	Learner type	Student number	Mean	Std. deviation	Mean of std. error
E1adj	Foreign learner	26	6.35	3.622	0.710
	Chinese learner	26	7.88	3.514	0.689
E1n	Foreign learner	26	4.62	2.418	0.474
	Chinese learner	26	4.23	1.840	0.361
E1v	Foreign learner	26	4.42	2.580	0.506
	Chinese learner	26	4.04	2.010	0.394
E3adj	Foreign learner	26	8.85	2.572	0.504
	Chinese learner	26	9.42	1.629	0.319
E3n	Foreign learner	26	9.81	0.981	0.192
	Chinese learner	26	9.04	2.457	0.482
E3v	Foreign learner	26	8.46	2.746	0.538
	Chinese learner	26	8.46	2.353	0.462
EF7adj	Foreign learner	26	7.31	3.234	0.634
	Chinese learner	26	7.31	3.803	0.746
E7n	Foreign learner	26	7.69	3.530	0.692
	Chinese learner	26	7.69	2.909	0.570
E7v	Foreign learner	26	8.08	3.486	0.684
	Chinese learner	26	8.85	2.572	0.504
E10adj	Foreign learner	26	9.23	1.840	0.361
	Chinese learner	26	9.42	1.629	0.319
E10n	Foreign learner	26	9.04	2.010	0.394
	Chinese learner	26	9.23	1.840	0.361
E10v	Foreign learner	26	9.23	1.840	0.361
	Chinese learner	26	9.04	2.010	0.394

Number (1,3,7,10) = frequency (1,3,7,10); E, English; adj., adjective; n, noun; v, verb.

From the perspective of the acquisition effect of artificial words, in exposure frequency 1, foreign students have the best acquisition effect on adjectives (*M* = 8.08), and Chinese learners have the worst acquisition effect on nouns and verbs (*M* = 6.5). In terms of the acquisition result of three different parts of speech in exposure frequency 1, the acquisition rate of foreign learners is higher than that of Chinese learners. In exposure frequency 3, the acquisition effect on nouns by foreign students is the best (*M* = 9.23), and on verbs is the worst (*M* = 6.92), and the average of verb acquisition of Chinese students and that of foreign students is the same. From the view of the overall effect of exposure frequency 3, foreign learners show higher acquisition of adjectives and nouns and are commensurate with Chinese students in the acquisition of verbs. In the acquisition of artificial words of exposure frequency 7, the acquisition effect on adjectives by foreign students is the best (*M* = 9.62), and on adjectives and verbs by Chinese students is the worst (*M* = 8.46). From the general situation of part-of-speech acquisition of artificial words of exposure frequency 7, the average acquisition rate of foreign students is higher than that of Chinese students. From the acquisition of artificial words of exposure frequency 10, foreign and Chinese students have the best acquisition effect on nouns and the former on verbs (the mean of the three is the same, *M* = 9.04), and Chinese learners have the worst acquisition effect on verbs (*M* = 8.27). In the 10-time exposure frequency condition, the difference in the acquisition rate of different vocabularies by Chinese and foreign learners is smaller than that when the exposure frequency is 3 and 7.

From the perspective of the overall effect of the acquisition rate of artificial words, frequency is still a major factor that leads to acquisition differences. With the increase in exposure frequency, the acquisition rate of words of all the parts of speech increases. However, under the same frequencies, the acquisition rate of foreign students is generally higher than that of Chinese students (except at frequency 10, for the acquisition of adjectives, the mean was 8.46 of the acquisition rate of foreign students and 8.65 of Chinese students). In terms of the acquisition rate of the part of speech, the acquisition rate of adjectives and nouns is high, and that of verbs is low, but such a difference decreases with the increase in exposure frequencies.

Meanwhile, the data indicate that the common characteristic of Chinese and foreign learners in the acquisition of artificial words was that the acquisition level of all the words was higher than 50%. The acquisition of adjectives at exposure frequency 7 by foreign learners was the highest (*M* = 9.62), and that of nouns and verbs at exposure frequency 1 by Chinese learners was the lowest (*M* = 6.45). Even the lowest acquisition rate was 10.45% higher than chance. This result proves that, regardless of the native language family of Chinese and foreign learners, in the sentence context, a similar effect exists in the exposure frequency of their acquisition of artificial words.

Table 2 below shows the acquisition of English words of the subjects. At exposure frequency 1, the acquisition of adjectives by Chinese students was the best (*M* = 7.88), and for verbs, it was the worst (*M* = 4.04). From the acquisition result of three parts of speech at exposure frequency 1, the acquisition rate of nouns and verbs by foreign students was higher than that of Chinese students, while for the acquisition rate of adjectives, Chinese students were higher than foreign students. At exposure frequency 3, the acquisition of nouns by foreign students was the best (*M* = 9.81) and poorest for verbs (*M* = 8.46), identical to the Chinese students. In terms of the general effect at exposure frequency 3, foreign learners and Chinese learners showed improvements in the acquisition of nouns and adjectives (*M* = 9.42 for the latter), while the acquisition level of verbs was equal for both. For the acquisition of English words at an exposure frequency of 7, the acquisition of verbs by Chinese students was the best (*M* = 8.85) and lowest for adjectives (*M* = 7.31 for both subject groups). Identification of part of speech and meaning of English vocabulary at exposure frequency 7 proved highest for verbs, then nouns, followed by adjectives. Here, the subject groups differed only slightly. The identification of verbs by Chinese students (*M* = 8.85) was slightly higher than that of foreign students (*M* = 8.08) and that of adjectives and nouns by both were the same (*M* = 7.31 for adjectives, *M* = 7.69 for nouns). In the acquisition of English words at an exposure frequency of 10, the performance with adjectives by Chinese students was the best (*M* = 9.42), with the lowest score demonstrated by Chinese students on verbs (*M* = 8.27) and by foreign learners on adj. (*M* = 8.46). Similar to the results for artificial words, the performance differences at an exposure frequency of 10 on the parts of the speech task by Chinese and foreign learners are smaller than those at exposure frequencies of 7 and 3.

Regarding the overall results with English vocabulary, frequency is still a major factor that influences task performance. With the increase in exposure frequency, the performance with words from all parts of speech increases. However, under the same frequencies, the acquisition rate of foreign students in low-frequency exposure (such as frequency 1) is generally higher than that of Chinese students. With increasing frequency, the difference between them decreases. The growth is not linear, but at frequency 7, the acquisition rate fell back, and the overall acquisition rate was lower than that at frequency 3. At an exposure frequency of 10, the acquisition rate is largely improved again. In terms of the identification of the part of speech (except for the verbs at frequency 7, *M* = 8.85 for Chinese students and *M* = 8.08 for foreign students), the identification of adjectives and nouns was high and that of verbs was low, but such a difference decreased with increasing exposure frequencies.

Similar to the acquisition of meaning and grammar of artificial words, the universality of the frequency effect that transcends the native language family at statistical significance is also generated during the acquisition of meaning and grammar of English words. Frequency has a universal effect beyond the level of chance for both foreign learners and Chinese learners. Although the data show that the acquisition rate of nouns and verbs of Chinese and foreign learners on single exposure is low (40.4–46.2%), this does not mean that the word form of language type leads to low acquisition of grammar and meaning of words only by Chinese learners, since the acquisition rate of both Chinese learners and foreign learners is similarly low. In contrast, it might properly indicate that low exposure frequency has no significant effect on the acquisition of meaning and grammar of any word forms regardless of whether they are artificial or English.

To learn the relation between experimental factors and accurately understand the influence of frequency on the identification of different parts of speech of artificial words by Chinese and foreign learners, the researcher conducted a repeated measures variance analysis, as shown in Table 3. Four independent variables are involved in this experiment, including the learner, language type, part of speech and frequency. The dependent variable is the score for word recognition.

**TABLE 3:** Variance analysis of repeated measurement of grammar and meaning of artificial words.

Source	Type III sum of squares	df	Mean square	*F*	Sig.
Language	28.926	1	28.926	2.023	0.161
Language × learner type	54.167	1	54.167	3.789	0.057
Frequency	1362.901	3	454.300	53.491	0.000
Frequency × learner type	11.058	3	3.686	0.434	0.729
Part of speech	60.136	2	30.068	6.953	0.001
Part of speech × learner type	9.495	2	4.748	1.098	0.338
Language × frequency	521.554	3	173.851	20.537	0.000
Language × frequency × learner type	10.737	3	3.579	0.423	0.737
Language × part of speech	30.088	2	15.044	3.364	0.039
Frequency × part of speech	205.248	6	34.208	6.265	0.000
Frequency × part of speech × learner type	46.274	6	7.712	1.412	0.209
Language × frequency × part of speech	112.220	6	18.703	4.000	0.001
Language × frequency × part of speech × learner type	20.393	6	3.399	0.727	0.628
Learner type	25.962	1	25.962	0.475	0.494

Significance value < 0.05.

The data (see Table 3) show that the main effect on frequency is significant (*F* = 53.491; *p* = 0.000 < 0.05), and the main effect of part of speech is significant (*F* = 6.953; *p* = 0.001 < 0.05). While a significant interaction exists among language * frequency (*F* = 20.537; *p* = 0.000 < 0.05), language * part of speech (*F* = 3.364; *p* = 0.039 < 0.05), frequency * part of speech (*F* = 6.265; *p* = 0.000 < 0.05), and language * frequency * part of speech (*F* = 4.000; *p* = 0.001 < 0.05), namely, under their mutual action, significant differences in the overall acquisition rate of meaning and grammar of words are observed.

However, the data show that the main effects of language type (artificial words and English) (*F* = 2.023; *P* = 0.161) and learner type (Chinese and foreign learners) (*F* = 0.475; *P* = 0.494) are insignificant, suggesting that the difference in the acquisition of grammar and meaning of artificial words and English at low frequencies by Chinese and foreign learners is not apparent, indicating that more similarity and consistency are reflected between them.

To determine the detailed differences in acquisition between the artificial words and English words, the present study performed a descriptive analysis (see Table 4) and paired-*T* test (Table not presented) on the frequency effect on the acquisition of artificial words and English words.

**TABLE 4:** Comparison between acquisition of artificial and English words.

Dep. variable: score				
Languages	Frequency	Mean	Std. deviation	Case
Artificial	1.00	7.1617	0.40212	6
	3.00	7.6000	0.82149	6
	7.00	8.9217	0.44634	6
	10.00	8.7450	0.31998	6
	Total	8.1071	0.90935	24
English	1.00	5.2583	1.53150	6
	3.00	8.9967	0.54010	6
	7.00	7.8317	0.56623	6
	10.00	9.2350	0.10114	6
	Total	7.8304	1.80014	24

The result demonstrates that the average score of the acquisition of artificial words (*M* = 8.1071) is higher than that of English words (*M* = 7.8304). The highest score occurs in frequency 10 for English words, while the lowest score occurs in frequency 1 for English words. The paired-*T* test result (*T* = 0.370; *P* = 0.736 > 0.05; two-tailed) indicates that there is no significant difference between artificial word acquisition and English word acquisition among Chinese and foreign learners.

## Discussion

From the experimental results, we find that the acquisition of grammar and meaning of artificial words by Chinese and foreign learners is complex and influenced by multiple factors. According to usage-based theory (Bybee, 2006; Tyler, 2010; Wang, 2011), any real language is used in a context and affected by the factors in the context. Language system and language competence base fundamentally on the use of language, and language system is exemplar-based and is gradually formed by learners’ frequent exposure to real communication situations. Besides, embodied cognitive linguistics emphasizes the unified influence of human brain, human body and the environment toward language acquisition. Learners’ culture could be one of the factors, which implicitly affects SLA.

In this section, we discuss the experimental results in relation to the four questions that the current study set out to address.

*RQ1. Do Chinese and foreign learners of English differ in the acquisition of grammar and meaning of artificial words and English words?*

The descriptive statistics for Chinese and foreign learners’ acquisition of meaning and grammar of artificial words, variance analysis result (main effect of Chinese and foreign learners is insignificant), and multiple comparisons for frequency effect show that there is no significant difference in the acquisition of grammar and meaning of artificial words between both types of learners. This indicates that Chinese and foreign learners only differ slightly in the acquisition of the part of speech and meaning of artificial words of alphabetic writing, which is distinct from the result of Chen et al.’s (2020) study of word form acquisition of artificial words of Chinese and foreign learners. However, as a whole, foreign learners’ acquisition of vocabulary at different exposure frequencies is better than that of Chinese learners.

Regarding the reasons why there are few significant differences between them in the acquisition of grammar and meaning of artificial words, we believe that although artificial words are closer to English words with regard to word form, compared with the meaning and grammar of target words, the difference between the two languages on word form is more obvious, and meaning may be interlinked or similar in the native language of Chinese and foreign learners. Furthermore, in this experiment, the target words are tested in the context of sentences, which means that the subjects (either Chinese learners or foreign learners) have more clues for obtaining knowledge of the target word than in the context of a single word, as Yang and Zhang (2021) indicated that frequency is sometimes embodied in one’s world knowledge and is the result of one’s past experience. Concerning the reason why the acquisition of the part of speech and meaning of artificial words by foreign learners is generally better than that of Chinese learners, we think it might be because the word form structure of artificial words is more similar to that of foreign learners’ L1, which can make foreign learners pay less attention to the processing of word forms, while more attention resources can be used for grammatical and semantic recognition of words. Chinese learners of hieroglyphs do not have such cognitive prerequisites. The data reflect that with the increase in acquisition frequency, the difference between them continually decreases. This means that when learners of different types of native language are exposed to words frequently, their competence for grammar and competence for meaning converge.

Based on the experimental result, we may infer that for the acquisition of words in a new language, in the initial stage, the more similar the word forms are, the better the acquisition effect is. The similarity of word form is a major cause that leads to rapid mastering of language for learners of alphabetic writing. However, with the extension of learning time and the increase in exposure frequency, the gap between them will narrow. Hieroglyphic learners might speed up the acquisition of new languages after they adapt to them and integrate the new languages into their own language system, including the meaning and grammar of new languages.

Another important finding in artificial word acquisition is that, for Chinese learners whose character pattern of their L1 is obviously different from alphabetic writing, the impact of the exposure frequency on the acquisition of grammar and meaning of vocabulary has reached the level of significance, indicating that the effect of frequency has transcended language types and has similar functions to the grammar and meaning acquisition of vocabulary of different language types.

The acquisition data of grammar and meaning of English words of Chinese and foreign learners are more complex. In terms of the exposure frequency and acquisition rate, the characteristics of fluctuation and change are shown. The English vocabulary acquisition rate of the three parts of speech at exposure frequency 1 is generally low; it increases greatly at frequency 3, decreases at exposure frequency 7, and increases obviously at frequency 10 again. The non-linear learning curve reflects what is seen in reality. After acquiring substantial new knowledge, learners begin to internalize and reconstruct knowledge in the brain, compare and assimilate with existing knowledge, and then acquire new knowledge after the reconstruction.

In terms of the acquisition of words of different parts of speech, at the same exposure frequency, the acquisition of adjectives is the best, followed by nouns and then verbs. This is similar to the phenomenon observed in daily teaching, in which performance with adjectives and nouns is better than with verbs. This result is very similar to Horst et al.’s (1998) finding that notional word acquisition had a higher acquisition score, and the images had a significant effect on the acquisition. This might also be related to the easier identification of the meaning of adjectives and nouns. As this experiment only involves the grammatical characteristics of the part of speech of words, other than the number, case, tense and voice of words, the influence of the saliency of word meaning on word acquisition is easier to show.

From the perspective of learners, the data of acquisition of English words show that the acquisition similarity of the grammar and meaning is larger than the difference of Chinese and foreign learners. This might explain that even though the word form of learners’ L1 is different, when two groups of learners learn a language at the same time for a period of time, they will no longer be influenced by the word form, grammar and meaning of their L1 and show more similarities in the acquisition of the target language. This point of view is also confirmed in Table 2 by the result of Chinese and foreign learners’ acquisition of different parts of speech at different frequencies. According to embodied theories (Boden, 2006; Wang, 2008; Atkinson, 2010), we assume that during the learning process the target culture embedded in the target language increasingly enhanced its effect toward the learners’ (Chinese or foreign) acquisition and constantly interact with learners’ cognition, and as a result, the acquisition achievement of Chinese learners and foreign learners tends to assimilate.

The data in Table 2 are in line with the results presented in Table 1 that Chinese and foreign learners have achieved good results in terms of the exposure frequency of artificial words and revealed the same characteristics of Chinese and foreign learners that the acquisition rate for the words with the same part of speech by both learners is low at exposure frequency 1. More importantly, these characteristics are shown in the real language–the acquisition of English vocabulary. This further proves that the exposure frequency plays an important role for Chinese learners who use non-alphabetic writing, and the exposure frequency surpasses the language family of its L1 and has similar functions to lexical acquisition.

From the analysis of RQ1, it is found that at the initial period of vocabulary acquisition, effect of language types embedded with native cultural factor for language acquisition is obvious. But as learners expose them more to vocabulary, this effect tends to fade, and the effect of frequency increases. According to embodied philosophy and usage-based theory, we may assume that native context is always the first factor exerting important influence on SLA. Nevertheless, this situation may change with the change of relevant factors. In this study, frequency effect shows its great increasing impact on SLA. This finding is supported by Ament et al.’s (2020) study which suggested that EMI, with an emphasis on both context effect and frequency effect, plays an important role in facilitating the acquisition of some functions of PMs. In the present study, it is also found that the cultural factor, specifically the writing of native language in this study, changes while learners learning a new type of writing. With increasing exposures, the frequency effect becomes the most prominent factor in SLA. On one hand, as Chinese learners learn a language of alphabetic writing, they may be influenced directly by the obvious difference of the writing at the beginning of the learning; on the other hand, when learners of different native language learn a same language, the culture influence tends to converge and the influence of new language will gradually overpass the influence of the original languages. During this period, effect of exposure frequency becomes the key factor.

*RQ2. What is the general role of frequency in Chinese and foreign learners’ acquisition of grammar and meaning of artificial and English words?*

In general, frequency has a prominent effect on the acquisition of grammar and meaning of artificial words and English words, indicating that the change of exposure frequency will result in acquisition change, the higher the frequency of exposure, the better the acquisition of grammar and meaning. This is supported by Qi and Wang’s (2020) study, which, based on the viewpoint of a usage-based approach, explored how input frequency and semantic feature affect language acquisition device and showed how with the increasing contact with specific language structure, learners gradually extract language use rules from these language constructs and establish the mapping relationship between structure and meaning in the brain. Therefore, frequency is the fundamental mechanism of language acquisition.

However, in this regard, the acquisition of artificial words and English words is differential. With the increase of frequency, the acquisition rate of artificial words increases by and large, but the increase is first fast and then slow, and the difference at frequency 7 and frequency 10 is not obvious. This shows that with grammar and meaning of artificial words, the function of frequency is obvious in the first stage, but with the increase in frequency, the growth of acquisition slows down. We can deduce that for grammar and meaning acquisition of artificial words, the plateau phenomenon of exposure frequency might exist, demonstrating that after a certain exposure frequency is reached, new acquisition becomes difficult.

For the acquisition of grammar and meaning of English words, the function of frequency fluctuates, and such fluctuations might still exist in the relationship between subsequent frequency increase and lexical acquisition rate, which differs from the frequency effect on that of artificial words. This might suggest that the frequency effect on grammar and meaning acquisition of vocabulary in real languages is more inclined to vary due to the influences of implicit culture and meaning, and for artificial languages, with no possibilities of being embedded with cultural elements, the effect of frequency tends to increase linearly. The analysis of RQ 2 proves that language could not be separated from its culture and environment. All the factors could explicitly or implicitly influence the acquisition of language, thus make the results irregular.

*RQ3. Does the interaction of factors such as learner type, language type, frequency and part of speech influence the acquisition of grammar and meaning of vocabulary?*

From the results of the multivariate analysis of Chinese and foreign learners’ acquisition of grammar and meaning of words, the frequency and part of speech are seen to be important factors that cause significant differences in vocabulary acquisition. This suggests that frequency is an important factor in promoting the acquisition of artificial words and English in general. As Larsen-Freeman (1976) indicated, frequency may be the only important factor that leads to acquisition change. It seems that in any language, the identification of parts of speech varies largely, but the common thing is that performance with adjectives and nouns is generally better than with verbs, and at the same time, it relates to the salience of words of different parts of speech and memorability of such words.

Regarding the combined effect of various factors on vocabulary acquisition, significance exists in four types of interactions. From the effects of the interaction of different factors, we find that language types, frequency and part of speech of a word are the three factors that can usually combine to cause significant differences in the acquisition of grammar and meaning of words, which indicates that to obtain good results in the acquisition of grammar and meaning, in addition to some single factors, such as frequency and part of speech, the combination of these factors can also play a very important role. This finding further supports Zhang and Fang’s (2020) study on frequency effect on collocation, which showed that language proficiency, constituent word frequency, lexical frequency are all factors influencing the acquisition of collocation. Second language learners need to infinitely approach the overall processing of collocations in native language to acquire the collocations. Frequency effect is a gradual transition and evolution from dependence on rules to overall synthesis as the input frequency increases and NNSs’ language proficiency improves.

The choice of the four factors of learner type, language type, frequency and part of speech as the variables for interaction analysis reflect our assumption that they might well represent the key elements in language acquisition: learner, language (embedded in culture), language use, and language system. The interaction of these factors is supposed to well present how language is processed in the real context. This in fact affirms the Inseparability Principle by Atkinson (2010) that mind, body, and world work together in SLA.

*RQ4 Does the acquisition of grammar and meaning of words vary in accordance to the difference of language type?*

As Table 4 shows, in general, the acquisition of grammar and meaning of artificial words is better than that of English words. However, the trend is not regular, for which one level of frequency effect, such as frequency 1, might be better for artificial words, another level of frequency effect, such as frequency 3, is better for English words. The insignificance of the paired-*T* test of the two types of languages also indicates that the type difference of language does not cause the acquisition difference. From the analysis above, we might conclude that the type of language, whether artificial or real, is not the factor that can greatly affect the acquisition of grammar and meaning of a language. In other words, despite the different types of languages, learners tend to process their grammar and meaning in more or less the same way, which shows that human beings share more commonalities than differences in the use of languages in their language mechanism. These findings are very similar to the results of Perez-Paredes and Bueno-Alastuey’s (2019) study, revealing that although learners of different native languages vary in frequency in using certainty adverbs, NSs, and NNSs share more similarities than differences in language use. These findings also provide more evidences that exposure frequency and usage-based approaches have strong explanatory power for SLA (Bybee, 2006; Ellis and Larsen-Freeman, 2009; Ambridge et al., 2015; Patterson, 2021).

What we need to attend is that in this study, the language types refer to the two types of languages (artificial and real) under the same category of alphabetic writing, while in the study by Chen et al. (2020), the language types refer to a more general category of hieroglyphic Chinese and alphabetic Keki artificial language. Despite the differences in languages at micro-level or macro-level, the results of these two experiments show that the frequency effect can help to transcend language barriers to make language learners reach a similarly high level of acquisition of a word as native speakers.

### Implications

This study has the following pedagogical implications: (1) Exposure frequency is important for their SLA, regardless of learners’ native language. Therefore, teachers should take into account exposure frequency of vocabulary when designing teaching materials or creating teaching tasks; (2) Context embedded with the target language culture should be created to raise learners’ cultural awareness of target language and facilitate learners’ acquisition vocabulary; (3) Attention should be paid to the factors of language learners, their native languages, input frequency and grammar of words, as these factors may interact with each other to affect learners’ acquisition of second language vocabulary; (4) Different parts of speech of a word may be processed differently and take different amounts of time for acquisition. Therefore, teachers are suggested to attend to these differences and design appropriate tasks for the acquisition of different words.

### Limitations and further research recommendations

Limitations of this study should also be noted: (1) The sample size is relatively small which might affect the generalizability of the study. (2) We are mainly concerned with how the overall differences between the hieroglyphic writing and alphabetic writing and their influences toward the acquisition of meaning and grammar of words, assuming that alphabetic writing, whatever its native language is, will have the same influence on SLA. This assumption may ignore the individual traits of different alphabetic languages, which may vary in its effect during the process of SLA; (3) Embodied theory was quoted as an important theoretical basis in this study and culture was supposed to play an important role in affecting SLA. However, the exploration on how culture exactly works in affecting SLA and how it interacts with exposure frequency for SLA was not examined in this study; (4) This study is restricted to the investigation of vocabulary acquisition in sentence context. Future experiment could be designed for study at discourse level; (5) The measurement of acquisition of meaning and grammar relied on the same method of multiple choice. This might obscure their acquisition differences.

For future research, we suggest that comparative study of vocabulary acquisition be carried out between EFL learners of a specific country of native alphabetic writing language and native EFL Chinese learners. We also encourage researchers to carry out studies of this comparative type on the acquisition context of discourse level. New experiment paradigms of eye-tracking and ERP are recommended for exploring the nuances between the learners of different language types.

## Conclusion

The present study, as one of the few of its kind, sheds light on the frequency effect on grammar and meaning acquisition by learners of different language types. First, despite different language types of learners, regarding acquisition in its initial stage, the more similar the word forms are, the better the acquisition effect would be. After being frequently exposed to certain words, learners of different language types tend to converge in competence for grammar and meaning of the words. Secondly, learners of different language types share more similarities than differences for grammar and meaning acquisition. As second language learning progresses, target culture tends to enhance its effect toward the learners’ acquisition increasingly. Thirdly, learner types, language types, part of speech of a word and exposure frequency interact and have combining interaction effect toward the acquisition. Finally, the results of this experimental study suggest that exposure frequency could possibly be the determining factor in the acquisition of grammar and meaning of words. The effect of frequency might transcend language types and has similar functions to the grammar and meaning acquisition of vocabulary of different language types.

## Data availability statement

The raw data supporting the conclusions of this article will be made available by the authors, without undue reservation.

## Ethics statement

The study was approved by the Research Ethics Committee of South China Normal University, China where the first author completed his Ph.D. thesis (which the current manuscript is based on). The patients/participants provided their written informed consent to participate in this study.

## Author contributions

JZ led the research project and contributed to the research design, data collection, data analysis, manuscript drafting, and revising. JH analyzed the data, reviewed, and revised the manuscript. Both authors have approved the submission.
