# Supplementary material for: A comparative study of frequency effect on acquisition of grammar and meaning of words between Chinese and foreign learners of English language
Source: Front Psychol. 2023 Jul 25;14:1125483. doi: 10.3389/fpsyg.2023.1125483 (PMC10411516; doi:10.3389/fpsyg.2023.1125483)
Supplement: Supplementary file 1 [file Data_Sheet_1.docx]

**APPENDICES**

**Appendix 1：68 Keki Artificial Words（McCandliss，et al. 1997）**

| **68 Keki Words and Their English Meaning** | | | |
| --- | --- | --- | --- |
| **Keki Words** | **English** | **Keki Words** | **English** |
| bakso | table | menti | woman |
| beme | say | monde | come |
| binde | drive | muble | want |
| birno | fruit | mula | white |
| brope  | hit | nake | start |
| bune | stand | nembe | drink |
| dalko | letter | nosko | wall |
| diste | catch | noti | girl |
| dimo | soup | nusti | cat |
| drosto | food | pame | do |
| druka | Long，tall | penka | black |
| duko | window | plune | buy |
| dalko | letter | prano | car |
| famso | store | preto | fire |
| feno | ball | pule | hold，take |
| firne | write | raste | read |
| fune | give | sasi | dog |
| galto | pen | sluka | bad |
| gilki | bird | sopla | small |
| gonta | happy | sulo | male |
| iska | short | sumpe | throw |
| kale | sit | suta | angry |
| kezo | tree | tane | be |
| kisko | paper | tanti | man |
| klano | house | teka | good |
| klito | book | tibe | see |
| krule | eat | tilko | door |
| kunda | big | tonde | put |
| kurso | chair | trazo | pot |
| lanso | water | tuso | bread |
| leke | go，walk | zalo | hand |
| lompo | picture | zempo | cup |
| luti | boy | ziko | hat |
| mako | stick | zoke | break |

**Appendix 2：Artificial Target Words of Different frequency level**

**Target words of Frequency 1**

beme v. say

dalko n. letter

gonta adj. happy

plune v. buy

famso n. store

mula adj. white

**Target words of Frequency 3.**

fune v. give

muble v. want

drosto n. food

sasi n. dog

iska adj short

druka adj. long，tall

**Target words of Frequency 7**

membe v. drink

tonde v. put

dimo n soup

gonta adj happy

penka adj. black

ziko n. hat

**Target words of Frequency 10**

binde v. drive

kunda adj big

leke v. go，walk

teka adj. good

zempo n. cup

duko n. window

**Appendix 3：Low Frequency Target Words of Different Frequency Level**

**Target Words of Frequency 1**

1. decile adj. quiet and easily controlled
2. groats n. the hulled and crushed grain of oats，wheat，or certain other cereals
3. roily adj. （of a liquid） agitated vigorously； in a state of turbulence/
4. perfuse vt. force a fluid through （a body part or tissue）
5. casbah n. an older or native quarter of many cities in northern Africa； the quarter in which the citadel is located
6. baled v. make into a large bundle

**Target Words of Frequency 3**

1. askari n. （in East Africa） a soldier or policeman
2. krater n. a cup with two ears used in ancient Greece or Rome
3. hyoid adj. of or relating to the tongue bone
4. jazzed adj. excited or delighted

5. glissade v. slide in a standing or squatting position

6. blazon v. to be shown or written in a very noticeable way

**Target Words of Frequency 7**

1. crus n. the leg，esp. from the knee to the foot
2. bummed adj. of very poor quality；
3. orang n. large long-armed ape
4. volute adj. having the form of a volute； spiral
5. halloo v. urge on with shouts
6. instate v. to set or establish a rank or office

**Target Words of Frequency 10**

1. benne n. another name for sesame ，East Indian annual erect herb；
2. tanka n. a form of Japanese poetry；
3. oxidizing adj.  changes chemically because of the effect of oxygen on it.
4. walloping，adj. great，wonderful
5. surfeit v. exceed，supply more than needed
6. catnap v. sleep shortly

**Appendix 4：Test of Artificial Target Words of Different Frequency Level**

**Artificial Target Words of Frequency 1**

1 beme

He bemed again and again that he knew nothing about it.

A. said B. told C. smiled D. beauty

2.dalko

The old man asked his nephew to write a dalko to his grandson，who left two months ago.

A. paper B. letter C. doll D. song

3. gonta

She seems very gonta with the result，for that all what she can do.

A. sad B. happy C. greatly D. gone

4. plune

The young mum plan to plune many books to help her little child develop good habit of reading，

A. borrow B. buy C. back D. get

5. famso

The famso does not seem attractive though，it provides very basic supplies for residents around.

A. famous B. farmer C. store D. struggle

6. mula

He put some special liquid on the paper and two lines of words appeared on the plain and mula paper.

A. black B. red C. muddy D. white

**Artificial Target Words of Frequency 3**

1. fune

His grandmother funed him a special gift for his 18^th^ birthday.

A. made B. bought C. gave D. fortunate

2. muble

That is all what he mubled，he told the doctor.

A. had B. gave C. wanted D. much

3. drosto

The thin girl tried every means to survive in the mountain with little drosto and water.

A. food B. fruit C. drought D. hill

4. sasi

Wherever he goes the sasi will follow though he not young anymore.

A. boy B. son C. dog D. tall

5. iska

His speech is iska and powerful，which makes opponents（对手）frightened.

A. long B. short D. lovely D. spreading

6. druka

He never thinks that he can be a student of that university，for that seems to druka way to go.

A. hard B. special C. long D. easy

**Artificial Target Words of Frequency 7**

1. membe

It is quite necessary to membe more water in such hot weather.

A. swallow B. eat C. drink D. play with

2. tonde

The children are warned not to toned sharp things in the bag and take to the plane.

A. prepare B. put C. hide D. touch

3. dimo

This restaurant is well known for a kind of special dimo which is said inherited several centuries ago.

A. soup B. drink C. food D. menu

4.sopla

Sopla as the apples might be ，they are full of vitamins.

A. big C. bitter C. start D. small

5. penka

You can hardly recognize a penka goat at dark night.

A. white B. fat C. strong D. black

6. ziko

Her ziko looks so fashionable and elegant is designed by a well-known Chinese designer.

A. clothes B. hat C. shoes D. watch

**Artificial Target Words of Frequency 10**

1. binde

She has the license，but he still could not binde .

A. walk B. run C. teach D. drive

2. kunda

You can hardly imagine how kunda the cake is，it breaks Guinness World Records

A. small B. big C. sweet D. chocolate

3. leke

He tends to leke around the lake for hours whenever he has something to think.

A. walk B. run C. sleep D. play

4. teka

They are not professional，but their performance are teka，at least not bad as some people say.

A. bad B. enjoyable C. good C. profession

5. zempo

The old man always uses the same zempo for coffee，which was said to be inherited from his mother’s mother.

A. bowl B. cup C. glass D. special

6. duko

It is clear that nobody can go away from the duko，for this is 21th story.

A. stairs B. window C. lift D. fire

**Appendix 5：Test of Low Frequency Target Words of Different Frequency levels**

**Low Frequency Target words of Frequency 1**

1. docile

Camel —— docile， patient and working hard makes itself good friend of human being in desert area.

A. quiet and easily controlled B. strong C. struggle D. fierce

2. groats

On the top of the mountain，you may find that the land of groats extends as far as your eyes can see.

A. grains like wheat B. water C. road D. great

3.roily

He was roily and shouted at me for not informing him of the information on time

A. oily B. roar C. very angry and agitated D. ready

4. perfuse

This method and help to perfuse the ink tool for reuse

A. use B. perfect C. force a fluid through D. return

5. baled

His father got up early and baleded the goods and be ready for the market

A. begged B. between C. made into a large bundle D. prepared

6. casbah

The casbah seems very well maintained even after centuries of weathering.

A. castle in Africa B. cellar C. case D. catch

**Low Frequency Target words of Frequency 3**

1. Askari

The askari walked silently into the room without knowing that he was followed by others.

A. professor B. asking C. policeman D. artist

1. krater

Nobody knows how much the krater is exactly worthy of，thought everybody knows that it is priceless.

A. basket B. a cup with two ears D. create D. bell

1. hyoid

The dentist has been working on it for an hour but still could not find the reason which might cause damage to her hyoid bone.

A. of or relating to the tongue bone B. eye C. mouth D. eat

1. jazzed

The football fans were extremely jazzed when they knew that Messi would not miss the game.

A. sad B. jumped C. annoyed D. excited or delighted

5. glissade

The dancer glissaded but stood up immediately，which made the audience surprised.

A. flew B. slid in a standing or squatting position C. stopped D. glad

6. blazon

The students blazoned the poster，but few students paid attention to it

A. to be shown in a very noticeable way B. blazed

C. baked D. torn down

**Low Frequency Target words of Frequency 7**

1. crus

His crus was attacked by the mean dog，which made him very worried due to the lack of trustworthy vaccine （medicine）

A. the leg，from the knee to the foot B. face C. walk D. arm

1. bummed

He was very much bummed after he got to know that he miss the chance again.

A. annoyed B. busy C. better d. buy

1. orang

The orang is very friendly and clever. It can even count.

A. ape B. monkey C. dog D. orange

1. volute adj. having the form of a volute； spiral

The general trend for the local economy is up though volute.

A. low B. down C. spiral D. increase

1. halloo

“Come back” he hallooed to his dog，which ran after a baby dog.

A. dragged B. pulled C. smiled D. urge on with shouts

6. instate

He was instated as the commander，but he seems very worried due to the general war situation.

A. asked B. said C given D set a rank

**Low Frequency Target words of Frequency 10**

1. benne

Experts say benne cannot only be used for making food oil，but also very good for health.

A. another name for sesame B. bun C. beep D. bean

1. tanka

He is a well-known tanka writer in Japan，but little is known about in the States.

A. thesis B. letter C. tender D. a form of poetry

3. oxidizing

After being peeled（削皮），apples can easily be oxidizing.

A. chemically change because of the effect of oxygen on it. B. dark C. yellow D. growing

4. walloping

Apart from the normal things in life，the walloping thing in his life is watching football games.

A. boring B. wonderful C. ordinary D. wonder

5. surfeit

He tends to surfeit himself whenever he goes to buffet.

A. supply more than needed B. save C. sacrifice D. satisfactory

6. catnap

The old professor catnapped and continued his experiment though it was late at night.

A. drunk a cup of coffee B. slept shortly C. had a meal D. laughter
